# Alternative Splicing in Alzheimer’s Disease

**DOI:** 10.13188/2376-922X.1000010

**Published:** 2015-08-15

**Authors:** Julia E. Love, Eric J. Hayden, Troy T. Rohn

**Affiliations:** Department of Biological Sciences, Science Building, Boise State University, USA

**Keywords:** Alzheimer’s disease, Alternative splicing, RNA Sequencing, Amyloid precursor protein, Tau, Apo lipoprotein E4, Antisense oligonucleotides

## Abstract

Neurodegenerative diseases have a variety of different genes contributing to their underlying pathology. Unfortunately, for many of these diseases it is not clear how changes in gene expression affect pathology. Transcriptome analysis of neurodegenerative diseases using ribonucleic acid sequencing (RNA Seq) and real time quantitative polymerase chain reaction (RT-qPCR) provides for a platform to allow investigators to determine the contribution of various genes to the disease phenotype. In Alzheimer’s disease (AD) there are several candidate genes reported that may be associated with the underlying pathology and are, in addition, alternatively spliced. Thus, AD is an ideal disease to examine how alternative splicing may affect pathology. In this context, genes of particular interest to AD pathology include the amyloid precursor protein (*APP*), *TAU*, and apolipoprotein E (*APOE*). Here, we review the evidence of alternative splicing of these genes in normal and AD patients, and recent therapeutic approaches to control splicing.

## Introduction

### Alzheimer’s disease

Alzheimer’s disease (AD) is the most common cause of dementia in the U.S.A. and is characterized by a progressive decline in various cognitive functions [1]. Common cognitive impairments associated with AD are lowered performance in memory, attention, language, visuospatial skills, and in executing tasks that were previously performed with ease [2]. The neuropathology of AD is characterized by both the accumulation of toxic, extracellular beta-amyloid deposition and neurofibrillary tangles resulting from an accumulation of hyperphosphorylated *tau* protein [3]. Age is the most important risk factor in AD, but the decline in cognitive function is individual-specific and is influenced by environmental factors, individual experience, and genetic pre-disposition [4].

Genetic risk factors associated with early onset AD (manifestation of AD prior to age 60) typically involve mutations in the amyloid precursor protein (*APP*) gene, presenilin 1 (*PSEN1*) gene, and presenilin2 (*PSEN2*) gene [5]. These genes ultimately enhance beta amyloid peptide production [6]. Different genetic risk factors are associated with both sporadic and late onset AD (cases 65 and older). Candidate genes include *A2M* (encoding alpha-2-macroglobulin), *ABCA1* and *2* (encoding ATP-binding cassette transporters 1 and 2, respectively), *CLU* (encoding clusterin), *PICALM* (encoding the phosphatidylinositol binding clathrin assembly protein), *SORL1* (encoding sortilin-related receptor gene), and *TREM2* (triggering receptor expressed on myeloid cells 2) [7]. *APOE, APP*, and *tau* also have alleles associated with late onset AD and are of particular interest for this review because they show disease specific alternative splicing variants. In these genes, alternatively spliced variants also show different levels of protein expression, which may in turn have important effects upon protein aggregation [8–10]. Understanding varying gene expression could ultimately answer questions about AD pathogenesis and identify possible targets for disease treatment. As proteomic and transcriptomic technologies advance, there is now the potential to identify neurodegenerative-specific changes in postmortem brain tissues. Using these approaches could be useful in understanding protein aggregation in AD and the underlying pathology of AD, which is currently unresolved.

## Alternative Splicing in Alzheimer’s disease

### Regulators of alternative splicing

**Figure 1:**
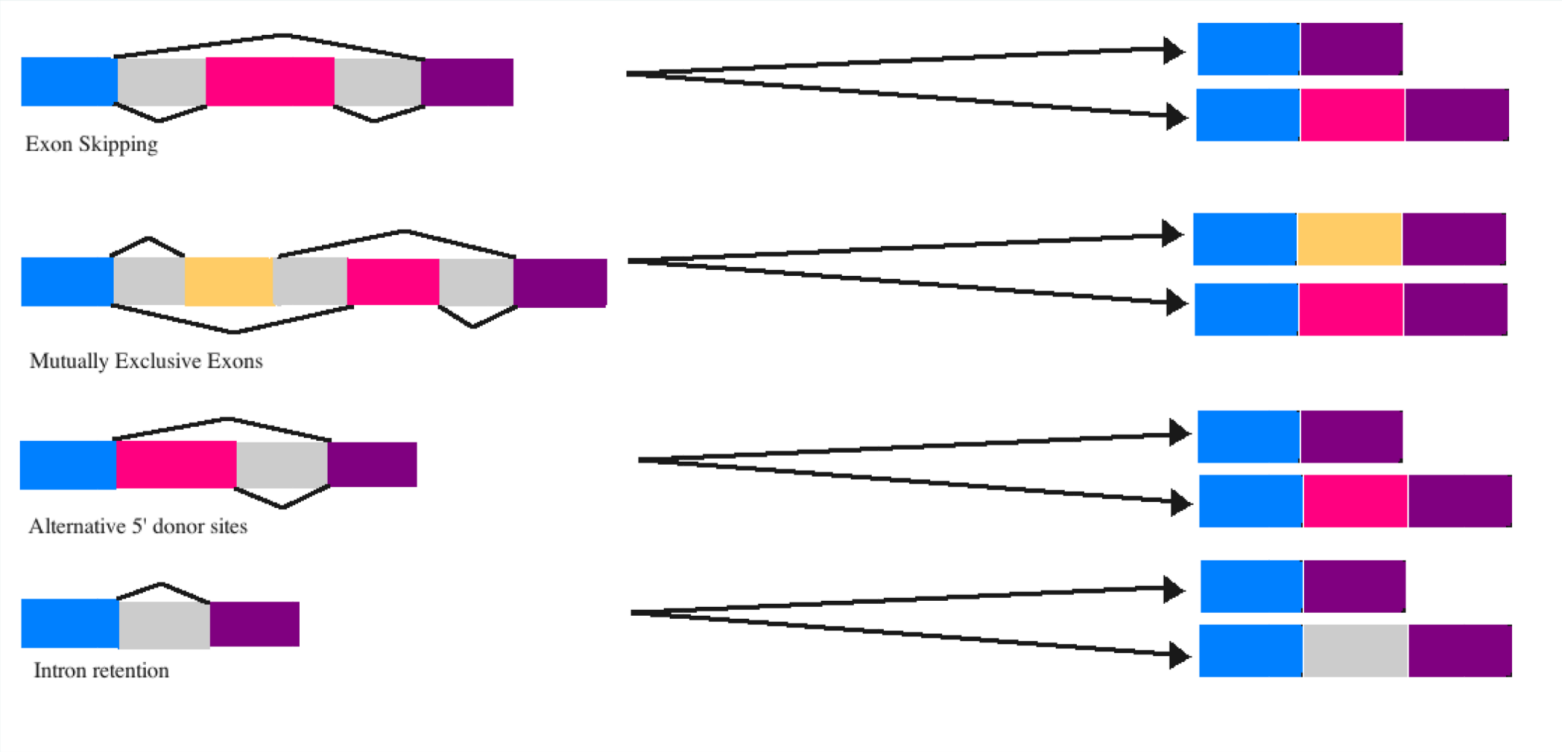
Alternative splicing patterns that may occur in AD The colored boxes are exons and gray boxes are introns. The black connection lines show the different patterns of including or excluding exons to generate different splicing products.

Alternative splicing is a main contributor to the complexity of organisms and their tissues. For example, in humans roughly 95% of multi-exonic genes are alternatively spliced resulting in 100,000 proteins in the human genome [11]. Alternative splicing occurs co- or post-transcriptionally resulting in multiple mRNA variants from a single gene (Figure 1). Alternative splicing is carried out by a spliceosome, which is made up of 5 small nuclear RNA (snRNA) molecules U1, U2, U4, U5, U6, and numerous proteins [9]. In addition, splicing is regulated by specific nucleotide sequences found within the mRNA (cis-elements). These elements include exonic splicing enhancers, exonic splicing silencers, and intronic splicing silencers [9]. In addition to cis-elements, trans-acting factors are a group of proteins that bind to cis-elements and are composed of serine and arginine rich (SR) proteins and heterogeneous nuclear ribonucleoproteins (hnRNPs). The presence of cis-elements and the tissue-specific expression of trans-acting factors regulate overall alternative splicing patterns [12]. Mutations in the spliceosomal machinery, cis-elements and trans-acting factors may contribute to the onset of disease. Several examples of the specific types of alternative splicing that may occur in AD are presented in Figure 1.

### Brain tissue specific alternative splicing

Alternative splicing is tissue specific, and especially important for brain tissue. The brain expresses more alternatively spliced genes than any other tissue according to current transcriptome analyses, a fact that likely contributes to the complexity of this organ [11]. Alternative splicing can be influenced by both the aging process and/ or environmental factors [13]. In AD, differential expression of genes and alternative splicing can potentially impact different signaling pathways. For example, genes that are over expressed in AD are genes associated with synaptogenesis, transmission, post-synaptic density, and long-term potentiation, all of which may contribute to disease pathogenesis associated with AD [12]. An example of up regulation of a gene in AD due to alternative splicing can be seen in immune-related pathways triggering increased neuro-inflammatory responses in the aging hippocampus [12]. On the other hand, it has been hypothesized that down regulation of specific genes in AD might lead to a compromise of DNA repair mechanisms consequently affecting chromosomal stability [10]. For example, over expression of amyloid-beta precursor protein-binding family B, member 2 (APBB2) can lead to cell cycle delays that cause down regulation of thymidilate synthase, an enzyme normally responsible for thymine formation. The decrease in thymine production in turn can lead to DNA damage and to a decrease in the ability to repair damaged DNA changing gene expression [14]. Changes in gene expression, either up or down in certain pathways supports the complexity of the role alternative splicing in AD (Table 1).

The application of transcriptome analysis in AD may provide insight into possible genes associated with the disease through RNA alternative splicing, antisense oligonucleotides, small nuclear ribonucleoproteins, and microRNAs. In this review, we will focus on what is known about normal and abnormal alternative splicing in some key genes associated with AD. Current research suggests that future transcriptome analysis will be important for determining how different splice variants could be used as diagnostic tools and targets for disease treatment.

## Alternative Splicing and β-amyloid Processing

Mutations that occur in *PSEN1, PSEN2*, and *APP* are most associated with early onset AD and affect the deposition of oligomeric β-amyloid peptides, which is the earliest known step in the disease pathology of AD [5,19]. More specifically, PSEN1 and PSEN2 are part of the gamma secretase complex and are involved in the cleavage of APP [5,19]. Incorrect cleavage of APP by the gamma secretase complex leads to the accumulation of toxic β-amyloid peptide [20]. Mutations found in *PSEN1* occur in intron 4 causing mis-splicing and exclusion of all or part of exon 4 [16]. In addition to this mis-splicing example, other mutations in *PSEN1* can lead to alterations in the expression of β-amyloid [21]. Specifically for *PSEN2*, a splice variant lacking exon 5 has been documented and is found in both early and late onset AD [22]. However, little is known about how this splice form contributes to disease pathology or progression.

By the use of ribonucleic acid sequencing (RNA-Seq), which quantifies the transcriptional outputs of both coding and non-coding RNA in the brain, there have been multiple transcription products presumed to be involved in the incorrect processing of APP [23]. Incorrect processing of APP leads to increased β-amyloid synthesis and accumulation. Alternative splicing occurring in these genes could be a key factor affecting this process. For example, RNA polymerase III has been proposed to transcribe a non-coding RNA that is responsible for exon 8 exclusion in amyloid-beta precursor protein-binding family B, member 2 (APBB2) [10]. APBB2 co-localizes with the amyloid intracellular c-terminal domain (AICD) of APP [24]. *APBB2* has three protein variants produced by alternative splicing (termed a,b, and c) and RNA polymerase III is responsible for producing more of the “a and b”exon 8 variants leading to exon 8 inclusion, while variant “c” results in exclusion of exon 8. The non-coding RNA transcribed by RNA polymerase III alters the ratio of alternative protein variants (a, b, and c) resulting in exon 8 inclusion and a reduction in the total amount of β-amyloid released [10]. Attempts to regulate alternative splicing to promote exon 8 inclusion underscores the potential of APBB2 as a target for treatment and prevention by decreasing β-amyloid release.

A second gene that contributes to β-amyloid aggregation is a group of proteins called RNA binding protein fork head box (RBFox). RBFox proteins are trans acting regulators of alternative splicing of the *APP* gene. The action of RBFox leads to the inclusion or exclusion of exon 7 within the *APP* gene. The inclusion of exon 7 is the dominant splice form in neural tissue of AD patients, and may contribute to β-amyloid production [25]. Moreover, *APP* iso-forms containing exon 7 are elevated in AD brain tissue and can activate the intracellular domain of *APP* as well as beta secretase [26]. Interestingly, the RBFox splice variant that retains exon 8 has also been thought to be involved with β-amyloid deposition in AD much like exon 7 [25]. There is still little known about factors contributing to exon 8 inclusion or exclusion, and further research is needed to examine the alternative splicing of the *APP* gene and the role that RBFox proteins play in this process. Elucidating the exact role that alternative splicing events play in enhancing the production of β-amyloid could uncover new drug targets for the treatment of AD.

A final example of an alternatively spliced gene resulting in incorrect APP processing and increased β-amyloid production is clusterin (*CLU*). Much like RBFox proteins, the function of clusterin and the exon variant associated with AD is relatively unclear [15]. In the experiment conducted by M. Szymanski et al. a single nucleotide polymorphism (SNP) in exon 1 promotes alternative splicing of a transcript of clusterin that enhances clusterin function and is associated with enhanced risk of AD [15]. Clusterin has been demonstrated to play a role in β-amyloid uptake and degradation [27], regulation of soluble β-amyloid levels across the blood-brain barrier [28], and binding with high affinity to soluble β-amyloid [29]. Although there is general uncertainly with regards to the mechanistic role of clusterin in AD pathology, there is direct evidence that clusterin modifies β-amyloid metabolism and/or deposition. This evidence comes from transgenic animal models comparing clusterin knockout mice with wild type mice [30]. Additional research on alterative splice variants of *CLU* could reveal the functional role of clusterin in AD. In addition, finding other alternatively spliced genes that affect either production of β-amyloid or its removal could provide valuable information on the underlying pathology associated with AD.

## Alternative Splicing of the Microtubule-Associated Protein, Tau in Alzheimer’s disease

A key step in the pathogenesis associated with AD is the post-translational modifications of *tau* including hyperphosphorylation, which leads to the formation of neurofibrillary tangles [31]. Functionally, *tau* is characterized as a microtubule-associated protein (MAP) and is important for stabilizing the cytoskeleton by binding to microtubules. Hyperphosphorylation of *tau* leads to a decrease binding affinity to microtubules and subsequent self-aggregation of *tau* into beta-sheet structures termed paired-helical filaments [31]. Exons 2, 3 and 10 of the *Tau* gene are alternatively spliced resulting in six known isoforms of *Tau* expressed in the brain [17]. Exon 10 encodes for the second microtubule-binding repeat and the inclusion of exon 10 generates *tau* isoforms with either three or four microtubule-binding sites (referred to as 3R-*tau* or 4R *tau*) [18]. The inclusion of exon 10 produces *tau* isoforms that are in a balanced ratio (1:1) in adult human brains with 3R-*tau* being primarily produced during development and the 4R-*tau* isoforms being produced in adulthood [18].

Serine/arginine (SR) rich proteins are one family of splicing factors involved in the alternative splicing of *tau*. In this regard, one such SR protein is SC35, which promotes exon 10 inclusion by acting on a SC35-like enhancer at the 5’ end of the *tau* RNA transcript [32]. Interesting, the phosphorylation of SC35 by protein kinase A (PKA) prevents inclusion of exon 10 resulting in increased expression of the 3R-*tau* isoform [33]. An unbalanced ratio of 3R-*tau* to 4R-*tau* is caused by down regulating the PKA phosphorylation pathway, which in turn promotes exon 10 inclusion [33]. Previous studies have shown that inclusion of exon 10 generating the 4R-*tau* isoform can lead to enhanced neurofibrillary tangles and *tau* aggregation [18].

Previous studies have suggested that in AD there is a disproportionate level of the 3R-*tau* isoform compared to the 4R form and this could be a key factor driving the formation of *tau* into paired helical filaments (PHFs) [34,35]. Numerous other SR proteins have roles in regulating the alternative splicing of the *Tau* gene including SRSF1, SC35, SRSR6, and SRSF9 all of which promote exon 10 inclusion. Alternatively, the activity of SRSF3, SRSF4, SRSF7, and SRSF11 suppress inclusion of exon 10 [9]. The actions of PKA on various SR proteins supports the notion that this is a common mechanism to modulate alternative splicing events [9]. It is possible that exon 10 of the *Tau* gene is highly regulated by alternative splicing in order to maintain the proper balance between 3R-*tau* and 4R-*tau* isoforms. Because of the important role of phosphorylation in splice site selection in the *Tau* gene, it has been proposed that controlling phosphorylation could be the basis to develop new therapeutic opportunities [36].

## Alternative Splicing of the APOE4 gene in Alzheimer’s disease

*APOE4* is a well-studied protein because the inheritance of the *APOE4* allele represents the single greatest genetic risk factor for late-onset AD. The *APOE* gene is polymorphic in human populations, with three common alleles, termed E2, E3, and E4. Harboring the E2 allele is protective against onset, while the E3 allele is neutral in this regard. In contrast carrying the E4 allele increases the risk of developing AD 4–10 fold [37]. ApoE4 has been suggested to affect both β-amyloid and neurofibrillary tangle pathology in AD [38,39]. ApoE4 is a major cholesterol transporter in the brain and cholesterol rich membrane domains increase β-amyloid production by affecting β and γ-secretase complexes [40].

Recent studies have shown that the E4 isoform is more prone to proteolysis than other APOE isoforms and this could be the reason for the increase in AD risk through either a loss or gain of function (for recent review see [38]). Thus, proteolysis of apoE4 may lead to a loss of function in its ability to remove β-amyloid and to transport cholesterol [38]. On the other hand, apoE4 fragments from proteolysis could lead to a toxic gain of function. For example, cleavage of apoE4 produces N- and C-terminal fragments that are neurotoxic in nature [41–44]. Tolar et al. showed that not only is a 22 kDa N-terminal fragment of apoE4 neurotoxic, but that it is significantly more toxic than the same fragment derived from the E3 isoform [43]. Unfortunately, much is still not known about the mechanism by which ApoE4 contributes to the pathogenesis of AD.

According to a genome wide association study (GWAS) utilizing cerebrospinal fluid (CSF) from AD subjects, several single nucleotide polymorphisms (SNPs) associated with *APOE* gene region of the brain were also associated with *tau* and phosphorylated *tau* (p*tau*) levels in the CSF. When cerebrospinal fluid levels of β-amyloid 1–42 levels were analyzed together with *tau*/p*tau*, a significant correlation was found with SNPs of *APOE* gene [39]. The authors of this study suggested that apoE variants could be modulating β-amyloid 1–42 levels as well as *tau* pathology. It is known that SNPs located near splice sites have the ability to change the splicing pattern of a gene [45].

Alternative splicing and alternative transcriptional promoter choice produces three main isoforms of *APOE4* (*APOE4-001, -002*, and -*005*) [8]. *APOE-001* and *APOE-002* isoforms contain exon 1 while the *APOE-005* isoform is generated by a promoter upstream of exon 2 [8]. Conflicting results have been obtained on the predominance of one *APOE4* isoform over the others. For example, RNA-Seq experiments originally suggested that the *APOE4-005* transcript was up regulated in AD while the *APOE4-001* isoform was down regulated [8]. However, Mills et al. was unable to confirm these results via the use of real time-quantitative polymerase chain reaction (RT-qPCR) [46]. The reasoning behind these discrepancies are not known but may be due to the possibility that RNA samples being taken from different areas of the temporal lobe in the two studies showed differential expression. In addition, the sample size in the Mills et al. study was 14 temporal lobe samples, whereas in the Twine et al. study the sample size was 1 temporal lobe sample. The findings from a small sample size in the Twine et al. study may be leading to an exaggerated difference between these studies. Alternatively, it is possible that the AD cases used in the two studies were not comparable in terms of pathology [46]. Finally, due to the issue of heterogeneity of the disease process in AD patients, the progression of the disease most likely varies significantly between affected individuals expressing the *APOE4* allele. Therefore, although the data support the notion that *APOE4* is alternatively spiced additional studies are warranted to address the degree to which splice variant of *APOE4* is up- or down regulated in normal versus AD tissues and at different stages of the disease.

## Targeting RNA as a Possible Treatment Strategy in Alzheimer’s disease

### Antisense Oligonucleotides

A recent approach to developing RNA specific therapeutics involves the use of short strings of nucleic acids that base pair to a target RNA molecule, which are referred to generally as antisense oligonucleotides (ASO) [47]. Often, the backbone chemistry of ASOs is modified to either encourage degradation of RNA or keep RNA from being degraded [47]. In this manner, endogenous RNA transcripts can be manipulated in numerous ways leading to alternative splicing, translation inhibition, and microRNA hindrance [47]. ASOs used as drugs are most often employed in a manner as to change levels of a target gene, and modulating alternative splicing is emerging as one such approach [48]. As previously mentioned, the U1 snRNA molecule plays a key role in alternative splicing. U1 snRNA has an additional role to suppress the processing of premature cleavage and polyadenylation of RNA [49]. Polyadenylation is the addition of a poly (A) tail to mRNA at the 3’ end, which normally serves as a protective role preventing enzymatic degradation. When U1 was inhibited by an ASO (“morphlino”), premature cleavage and polyadenylation occurred resulting in increased activity of U1 snRNA [50]. Such a process effectively changed the alternative splicing pattern due to increased premature cleavage and polyadenylation [8]. These observations are suggestive of a loss of U1 snRNA function in the AD brain, which in turn could promote the alternative splicing of genes that contribute to the underlying pathology associated with this disease [50].

One advantage that ASOs have in terms of therapeutic agents is their ability to cross the blood brain barrier, a limiting factor for much CNS therapeutics. An example of how ASOs can be applied to AD treatment is targeting pathological *tau* in AD. In *TAU* knock-out animal models, β-amyloid-induced cognitive loss was restored by targeting ASOs to reduce the amount of *tau* protein [51]. Therefore, reducing *tau* protein levels could be a therapeutic technique achievable through ASOs [51]. In addition to *tau*, ASOs have also been used to lower β-amyloid loads in mutant mice over expressing human APP [52]. Following administration of ASOs, APP levels were reduced and learning and memory was rescued [52].

## Conclusion

Alternative splicing is an important post-transcriptional regulatory mechanism during gene expression that results in a single gene coding for multiple proteins. In AD, however, alternative splicing of the *APP*, *TAU*, or the *APOE4* gene may contribute to the disease pathology. Understanding the role of alternative splicing of AD-associated genes may not only shed light on the possible molecular mechanisms underlying this disease, but also for the pathology of other neurodegenerative diseases as well. As RNA-Seq technology continues to advance, it will become easier to analyze AD as well as other neurodegenerative diseases on a molecular level.

## Figures and Tables

**Table 1:** Alternatively spliced genes and their associated effects in AD.

Protein	Gene Affected	Exon Spliced	Observation	Method	Ref
**APBB2**	*APP*	Exon 8 Exclusion	Increase in beta-amyloid release	RT-qPCR	[10]
**RBFox**	*APP*	Exon 7 Exclusion	Increase in beta-amyloid production	RT-qPCR	[15]
**Presenilin 1**	*PSEN1*	Exon 4 Exclusion	Improper cleavage of APP increasing beta-amyloid deposition.	RT-qPCR	[16]
**Presenilin 2**	*PSEN2*	Exon 5 Exclusion	Improper cleavage of APP increasing beta-amyloid deposition.	RT-qPCR	[16]
**Apolipoprotein E**	*APOE*	Exon 5 Exclusion	Increase beta- amyloid deposition, and or affecting tau structure.	RNA-Seq	[11]
***Tau***	*MAPT*	Exon 10 Inclusion	Generating an imbalance of 3R-*tau* and 4R-*tau* leading to *tau* protein aggregation.	RT-qPCR	[13,17,18]

## References

[1] Cummings JL (2004). Alzheimer’s disease. N Engl J Med.

[2] Albert MS, DeKosky ST, Dickson D, Dubois B, Feldman HH (2011). The diagnosis of mild cognitive impairment due to
Alzheimer’s disease: recommendations from the National Institute on
Aging-Alzheimer’s Association workgroups on diagnostic guidelines
for Alzheimer’s disease. Alzheimers Dement.

[3] Ferreira ST, Klein WL (2011). The Aβ oligomer hypothesis for synapse failure and memory
loss in Alzheimer’s disease. Neurobiol Learn Mem.

[4] Fischer A (2014). Epigenetic memory: the Lamarckian brain. EMBO J.

[5] Schellenberg GD, Bird TD, Wijsman EM, Orr HT, Anderson L (1992). Genetic linkage evidence for a familial Alzheimer’s
disease locus on chromosome 14. Science.

[6] Vetrivel KS, Zhang YW, Xu H, Thinakaran G (2006). Pathological and physiological functions of
presenilins. Mol Neurodegener.

[7] Alonso Vilatela ME, Lopez-Lopez M, Yescas-Gomez P (2012). Genetics of Alzheimer’s disease. Arch Med Res.

[8] Twine NA, Janitz K, Wilkins MR, Janitz M (2011). Whole transcriptome sequencing reveals gene expression and
splicing differences in brain regions affected by Alzheimer’s
disease. PLoS One.

[9] Qian W, Liu F (2014). Regulation of alternative splicing of tau exon 10. Neurosci Bull.

[10] Penna I, Vassallo I, Nizzari M, Russo D, Costa D (2013). A novel snRNA-like transcript affects amyloidogenesis and cell
cycle progression through perturbation of Fe65L1 (APBB2) alternative
splicing. Biochim Biophys Acta.

[11] Mills JD, Nalpathamkalam T, Jacobs HI, Janitz C, Merico D (2013). RNA-Seq analysis of the parietal cortex in Alzheimer’s
disease reveals alternatively spliced isoforms related to lipid
metabolism. Neurosci Lett.

[12] Stilling RM, Benito E, Gertig M, Barth J, Capece V (2014). De-regulation of gene expression and alternative splicing affects
distinct cellular pathways in the aging hippocampus. Front Cell Neurosci.

[13] Karambataki M, Malousi A, Kouidou S (2014). Risk-associated coding synonymous SNPs in type 2 diabetes and
neurodegenerative diseases: genetic silence and the underrated association
with splicing regulation and epigenetics. Mutat Res.

[14] Bruni P, Minopoli G, Brancaccio T, Napolitano M, Faraonio R (2002). Fe65, a ligand of the Alzheimer’s beta-amyloid precursor
protein, blocks cell cycle progression by down-regulating thymidylate
synthase expression. J Biol Chem.

[15] Szymanski M, Wang R, Bassett SS, Avramopoulos D (2011). Alzheimer’s risk variants in the clusterin gene are
associated with alternative splicing. Transl Psychiatry.

[16] De Jonghe C, Cruts M, Rogaeva EA, Tysoe C, Singleton A (1999). Aberrant splicing in the presenilin-1 intron 4 mutation causes
presenile Alzheimer’s disease by increased Abeta42
secretion. Hum Mol Genet.

[17] Andreadis A, Brown WM, Kosik KS (1992). Structure and novel exons of the human tau gene. Biochemistry.

[18] Goedert M, Spillantini MG, Jakes R, Rutherford D, Crowther RA (1989). Multiple isoforms of human microtubule-associated protein tau:
sequences and localization in neurofibrillary tangles of Alzheimer’s
disease. Neuron.

[19] Levy-Lahad E, Wijsman EM, Nemens E, Anderson L, Goddard KA (1995). A familial Alzheimer’s disease locus on chromosome
1. Science.

[20] Baulac S, LaVoie MJ, Kimberly WT, Strahle J, Wolfe MS (2003). Functional gamma-secretase complex assembly in Golgi/trans-Golgi
network: interactions among presenilin, nicastrin, Aph1, Pen-2, and
gamma-secretase substrates. Neurobiol Dis.

[21] Cruts M, Van Broeckhoven C (1998). Presenilin mutations in Alzheimer’s
disease. Hum Mutat.

[22] Sato N, Hori O, Yamaguchi A, Lambert JC, Chartier-Harlin MC (1999). A novel presenilin-2 splice variant in human Alzheimer’s
disease brain tissue. J Neurochem.

[23] Sutherland GT, Janitz M, Kril JJ (2011). Understanding the pathogenesis of Alzheimer’s disease:
will RNA-Seq realize the promise of transcriptomics?. J Neurochem.

[24] Cao X, Sudhof TC (2004). Dissection of amyloid-beta precursor protein-dependent
transcriptional transactivation. J Biol Chem.

[25] Alam S, Suzuki H, Tsukahara T (2014). Alternative splicing regulation of APP exon 7 by RBFox
proteins. Neurochem Int.

[26] Belyaev ND, Kellett KA, Beckett C, Makova NZ, Revett TJ (2010). The transcriptionally active amyloid precursor protein (APP)
intracellular domain is preferentially produced from the 695 isoform of APP
in a {beta}-secretase-dependent pathway. J Biol Chem.

[27] Li X, Ma Y, Wei X, Li Y, Wu H (2014). Clusterin in Alzheimer’s disease: a player in the
biological behavior of amyloid-beta. Neurosci Bull.

[28] Zlokovic BV, Martel CL, Matsubara E, McComb JG, Zheng G (1996). Glycoprotein 330/megalin: probable role in receptor-mediated
transport of apolipoprotein J alone and in a complex with Alzheimer disease
amyloid beta at the blood-brain and blood-cerebrospinal fluid
barriers. Proc Natl Acad Sci U S A.

[29] Matsubara E, Frangione B, Ghiso J (1995). Characterization of apolipoprotein J-Alzheimer’s A beta
interaction. J Biol Chem.

[30] DeMattos RB, O’Dell MA, Parsadanian M, Taylor JW, Harmony JA (2002). Clusterin promotes amyloid plaque formation and is critical for
neuritic toxicity in a mouse model of Alzheimer’s
disease. Proc Natl Acad Sci U S A.

[31] Alonso A, Zaidi T, Novak M, Grundke-Iqbal I, Iqbal K (2001). Hyperphosphorylation induces self-assembly of tau into tangles of
paired helical filaments/straight filaments. Proc Natl Acad Sci U S A.

[32] Qian W, Liang H, Shi J, Jin N, Grundke-Iqbal I (2011). Regulation of the alternative splicing of tau exon 10 by SC35 and
Dyrk1A. Nucleic Acids Res.

[33] Chen C, Jin N, Qian W, Liu W, Tan X (2014). Cyclic AMP-dependent protein kinase enhances SC35-promoted Tau
exon 10 inclusion. Mol Neurobiol.

[34] Espinoza M, de Silva R, Dickson DW, Davies P (2008). Differential incorporation of tau isoforms in Alzheimer’s
disease. J Alzheimers Dis.

[35] Goedert M, Jakes R (2005). Mutations causing neurodegenerative tauopathies. Biochim Biophys Acta.

[36] Hartmann AM, Rujescu D, Giannakouros T, Nikolakaki E, Goedert M (2001). Regulation of alternative splicing of human tau exon 10 by
phosphorylation of splicing factors. Mol Cell Neurosci.

[37] Eisenstein M (2011). Genetics: finding risk factors. Nature.

[38] Rohn TT (2013). Proteolytic cleavage of apolipoprotein e4 as the keystone for the
heightened risk associated with Alzheimer’s disease. Int J Mol Sci.

[39] Cruchaga C, Kauwe JS, Harari O, Jin SC, Cai Y (2013). GWAS of cerebrospinal fluid tau levels identifies risk variants
for Alzheimer’s disease. Neuron.

[40] Ye S, Huang Y, Mullendorff K, Dong L, Giedt G (2005). Apolipoprotein (apo) E4 enhances amyloid beta peptide production
in cultured neuronal cells: apoE structure as a potential therapeutic
target. Proc Natl Acad Sci U S A.

[41] Huang Y, Liu XQ, Wyss-Coray T, Brecht WJ, Sanan DA (2001). Apolipoprotein E fragments present in Alzheimer’s disease
brains induce neurofibrillary tangle-like intracellular inclusions in
neurons. Proc Natl Acad Sci U S A.

[42] Chang S, ran Ma T, Miranda RD, Balestra ME, Mahley RW (2005). Lipid- and receptor-binding regions of apolipoprotein E4
fragments act in concert to cause mitochondrial dysfunction and
neurotoxicity. Proc Natl Acad Sci U S A.

[43] Tolar M, Marques MA, Harmony JA, Crutcher KA (1997). Neurotoxicity of the 22 kDa thrombin-cleavage fragment of
apolipoprotein E and related synthetic peptides is
receptor-mediated. J Neurosci.

[44] Andrews-Zwilling Y, Bien-Ly N, Xu Q, Li G, Bernardo A (2010). Apolipoprotein E4 causes age- and Tau-dependent impairment of
GABAergic interneurons, leading to learning and memory deficits in
mice. J Neurosci.

[45] Faber K, Glatting KH, Mueller PJ, Risch A, Hotz-Wagenblatt A (2011). Genome-wide prediction of splice-modifying SNPs in human genes
using a new analysis pipeline called AASsites. BMC Bioinformatics.

[46] Mills JD, Sheahan PJ, Lai D, Kril JJ, Janitz M (2014). The alternative splicing of the apolipoprotein E gene is
unperturbed in the brains of Alzheimer’s disease
patients. Mol Biol Rep.

[47] Gryaznov S, Skorski T, Cucco C, Nieborowska-Skorska M, Chiu CY (1996). Oligonucleotide N3’-->P5’
phosphoramidates as antisense agents. Nucleic Acids Res.

[48] DeVos SL, Miller TM (2013). Antisense oligonucleotides: treating neurodegeneration at the
level of RNA. Neurotherapeutics.

[49] Kaida D, Berg MG, Younis I, Kasim M, Singh LN (2010). U1 snRNP protects pre-mRNAs from premature cleavage and
polyadenylation. Nature.

[50] Bai B, Hales CM, Chen PC, Gozal Y, Dammer EB (2013). U1 small nuclear ribonucleoprotein complex and RNA splicing
alterations in Alzheimer’s disease. Proc Natl Acad Sci U S A.

[51] Leroy K, Ando K, Laporte V, Dedecker R, Suain V (2012). Lack of tau proteins rescues neuronal cell death and decreases
amyloidogenic processing of APP in APP/PS1 mice. Am J Pathol.

[52] Kumar VB, Farr SA, Flood JF, Kamlesh V, Franko M (2000). Site-directed antisense oligonucleotide decreases the expression
of amyloid precursor protein and reverses deficits in learning and memory in
aged SAMP8 mice. Peptides.

